# Author Correction: Pr and Pfr structures of plant phytochrome A

**DOI:** 10.1038/s41467-026-71456-2

**Published:** 2026-03-30

**Authors:** Soshichiro Nagano, David von Stetten, Kaoling Guan, Peng-Yuan Chen, Chen Song, Thomas Barends, Manfred S. Weiss, Christian G. Feiler, Katerina Dörner, Iñaki de Diego Martinez, Robin Schubert, Johan Bielecki, Lea Brings, Huijong Han, Konstantin Kharitonov, Chan Kim, Marco Kloos, Jayanath C. P. Koliyadu, Faisal H. M. Koua, Ekaterina Round, Abhisakh Sarma, Tokushi Sato, Christina Schmidt, Joana Valerio, Agnieszka Wrona, Joachim Schulz, Raphael de Wijn, Romain Letrun, Richard Bean, Adrian Mancuso, Karsten Heyne, Jon Hughes

**Affiliations:** 1 Institute for Plant Physiology, Justus Liebig University, Giessen, Germany; 2 Department of Physics, Free University of Berlin, Berlin, Germany; 3 Department of Biomolecular Mechanisms, Max Planck Institute for Medical Research, Heidelberg, Germany; 4 European Molecular Biology Laboratory (EMBL), Hamburg, Germany; 5 Department of Molecular Biosciences, University of Texas at Austin, Austin, Texas USA; 6 Department of Analytical Chemistry, University of Leipzig, Leipzig, Germany; 7 Helmholtz-Zentrum Berlin für Materialien und Energie, BESSY II, Macromolecular Crystallography, Berlin, Germany; 8 European XFEL GmbH, Schenefeld, Germany; 9 Diamond light source, Didcot, United Kingdom

**Keywords:** Light responses, Nanocrystallography

Correction to: *Nature Communications* 10.1038/s41467-025-60738-w, published online 21 June 2025

In this article Soshichiro Nagano should have been denoted as a corresponding author. The original article has been updated.

