# Supplementary material for: Pr and Pfr structures of plant phytochrome A
Source: Nat Commun. 2025 Jun 21;16:5319. doi: 10.1038/s41467-025-60738-w (PMC12182589; doi:10.1038/s41467-025-60738-w)
Supplement: Supplementary file 1 — Supplementary Information [file 41467_2025_60738_MOESM1_ESM.pdf]

Supplementary Information

Pr and Pfr structures of plant phytochrome A

Nagano *et al.*

List of contents

Supplementary Table:

1	Data collection and refinement statistics

Supplementary Figures:

1	Domain architecture
2	Pfr→Pr thermal reversion of PCB & PΦB adducts
3	Superimposed MX and SFX structures of chromophore and surrounding pocket of PCB & PΦB adducts of soybean phyA(nPAS-GAF) as Pr
4	Comparison of MX structures 8R44 & 8R45 (PCB & PΦB adducts as Pr)
5	TEM images and UV-Vis absorption spectra of microcrystals
6	Comparison of SFX datasets 9ER4 and replicate for Pr
7	Comparison of Pr 9ER4 *(*SFX) and 8R44 (MX)
8	Comparison of Pr chromophore pocket in the 8R44 (MX) and 9ER4 (SFX) structures
9	Evidence for Pr to Pfr conformational changes in the chromophore region from the q-weighted Fo(light)–Fo(dark) difference map
10	SFX data processing
11	2Fo-Fc electron density maps
12	Fo-Fc polder omit maps

Supplementary Discussion:

Dataset and structure comparisons

Supplementary Methods:

Calculation of difference- and extrapolated maps

Supplementary References

Supplementary Table 1 | Data collection and refinement statistics*

	9ER4	9F4I	8R44	8R45
**Protein**				
Ligand / State	PCB / Pr	PCB / Pfr	PCB / Pr	PΦB/ Pr
**Data collection**				
Facility / Beamline	EuXFEL / SPB/SFX	EuXFEL / SPB/SFX	BESSY II / MX14.3	BESSY II / MX14.2
Wavelength (Å)	1.33	1.33	0.90	0.92
No. crystals	61275	176334	1	1
Temperature (K)	294	294	50	100
Space group	$P\ 2_1$	$P\ 2_1$	$P\ 2_1$	$P\ 2_1$
Cell dimensions				
a, *b*, *c* (Å)	56.5, 115.1, 69.8	56.5, 115.1, 69.8	55.3, 111.8, 68.3	55.5, 112.5, 68.4
α, β, γ (°)	90, 92.68, 90	90, 92.7, 90	90, 92.3, 90	90, 92.1, 90
Resolution (Å)	20.49 - 2.20 (2.28 - 2.20)	20.79 - 2.20 (2.28 - 2.20)	43.8 - 1.58 (1.64 - 1.58)	42.34 - 1.86 (1.93 - 1.86)
R_{merge}			0.051 (1.058)	0.116 (2.270)
R_{pim}			0.036 (0.629)	0.046 (0.464)
R_{split}	0.1716 (0.9743)	0.1088 (1.0840)		
$I / \sigma I$	5.58 (0.96)	7.88 (0.71)	12.84 (1.07)	12.88 (1.70)
Completeness (%)	100 (100)	100 (100)	99.58 (99.73)	99.92 (99.99)
Redundancy	491.6 (371.1)	1282.2 (965.1)	3.7 (3.6)	6.9 (6.5)
**Refinement**				
Resolution (Å)	20.49 - 2.20 (2.28 - 2.20)	20.48 - 2.2 (2.25 - 2.20)	43.8 - 1.58 (1.64 - 1.58)	42.34 - 1.86 (1.93 - 1.86)
No. reflections	45171	45048	112709	70335
R_{work} / R_{free}	0.2409 / 0.2939	0.1907 / 0.2314	0.182/0.202	0.169/0.192
No. atoms				
Protein	4803	4648	4924	4984
Ligand/ion	86	86	158	99
Water	138	132	586	465
B-factors ($Å^2$)				
Protein	47.81	54.66	31.93	34.94
Ligand/ion	56.36	70.17	41.70	47.65
Water	45.21	49.07	37.16	37.64
R.m.s. deviations				
Bond lengths (Å)	0.010	0.010	0.012	0.011
Bond angles (°)	1.46	1.23	1.70	1.68
**Ramachandran**				
Favoured	98.50	95.70	98.86	97.75
Allowed	1.33	3.26	1.14	2.09
Disallowed	0.17	1.03	0	0.16

*Values in parentheses are for highest-resolution shell.

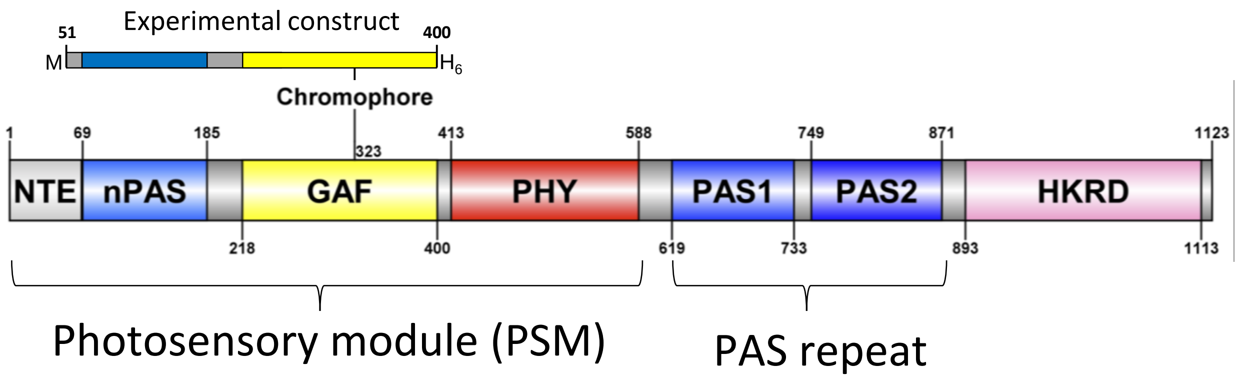

NTE	N-terminal extension
nPAS	N-terminal Per / ARNT / Sim domain
GAF	cGMP-specific phosphodiesterases / adenylyl cyclases / Fhl domain
PHY	Phytochrome-specific domain
PAS1/2	Per / ARNT / Sim domains 1 & 2
HKRD	Histidine kinase-related domain

Supplementary Fig. 1 | Domain architecture of soybean phytochrome A.

Residue numbers of domain boundaries and chromophore attachment site are shown. Domain acronyms are also explained.

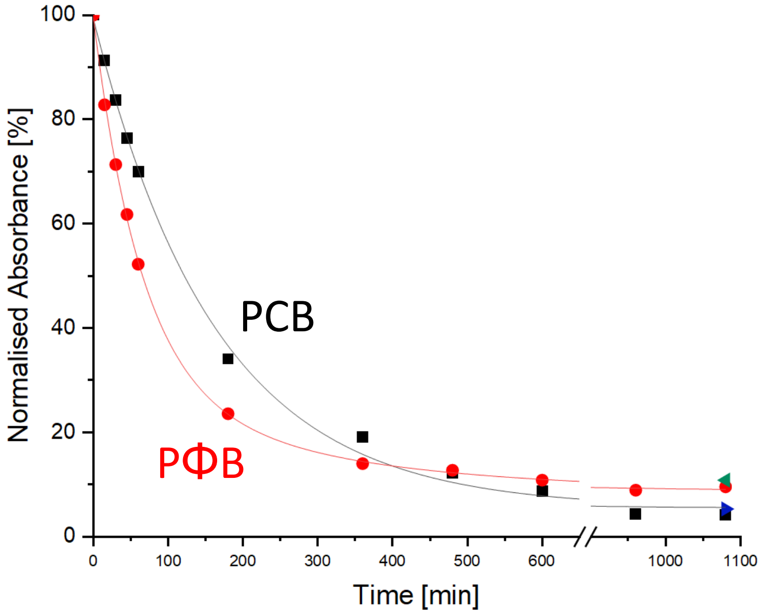

Supplementary Fig. 2 | Pfr→Pr thermal reversion in PCB & PΦB adducts of soybean phyA(nPAS-GAF). Pfr concentration in solution at pH 7.8 was monitored by absorption at 715 nm. The measuring light was extinguished between timepoints; measurement at the final timepoint was repeated for samples held in darkness throughout (triangles).

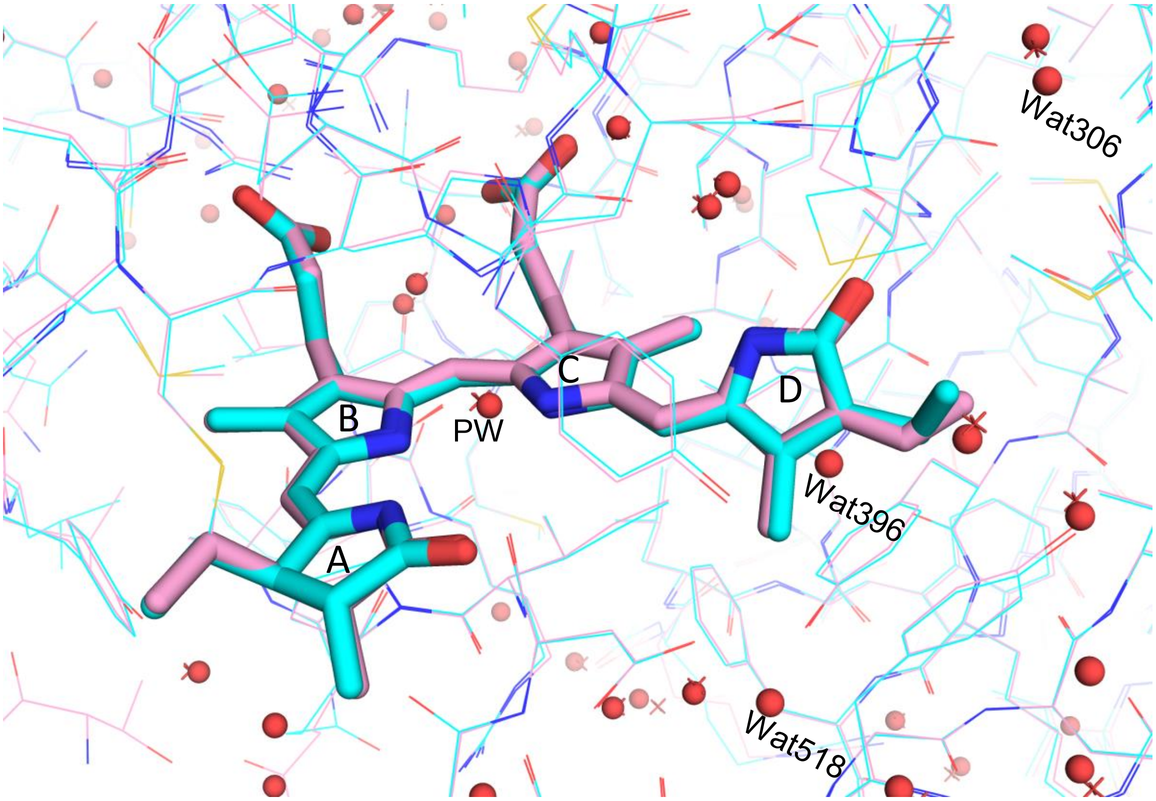

Supplementary Fig. 3 | Superimposed MX and SFX structures of chromophore and surrounding pocket of PCB & PΦB adducts of soybean phyA(nPAS-GAF) as Pr. B-chain carbons of 8R44 (PCB, cyan) and 8R45 (PΦB, pink) are shown. Waters are shown as red spheres for 8R44 and stars for 8R45. Waters 518, 396 and 306 in 8R44 are poorly supported by electron density in 8R45.

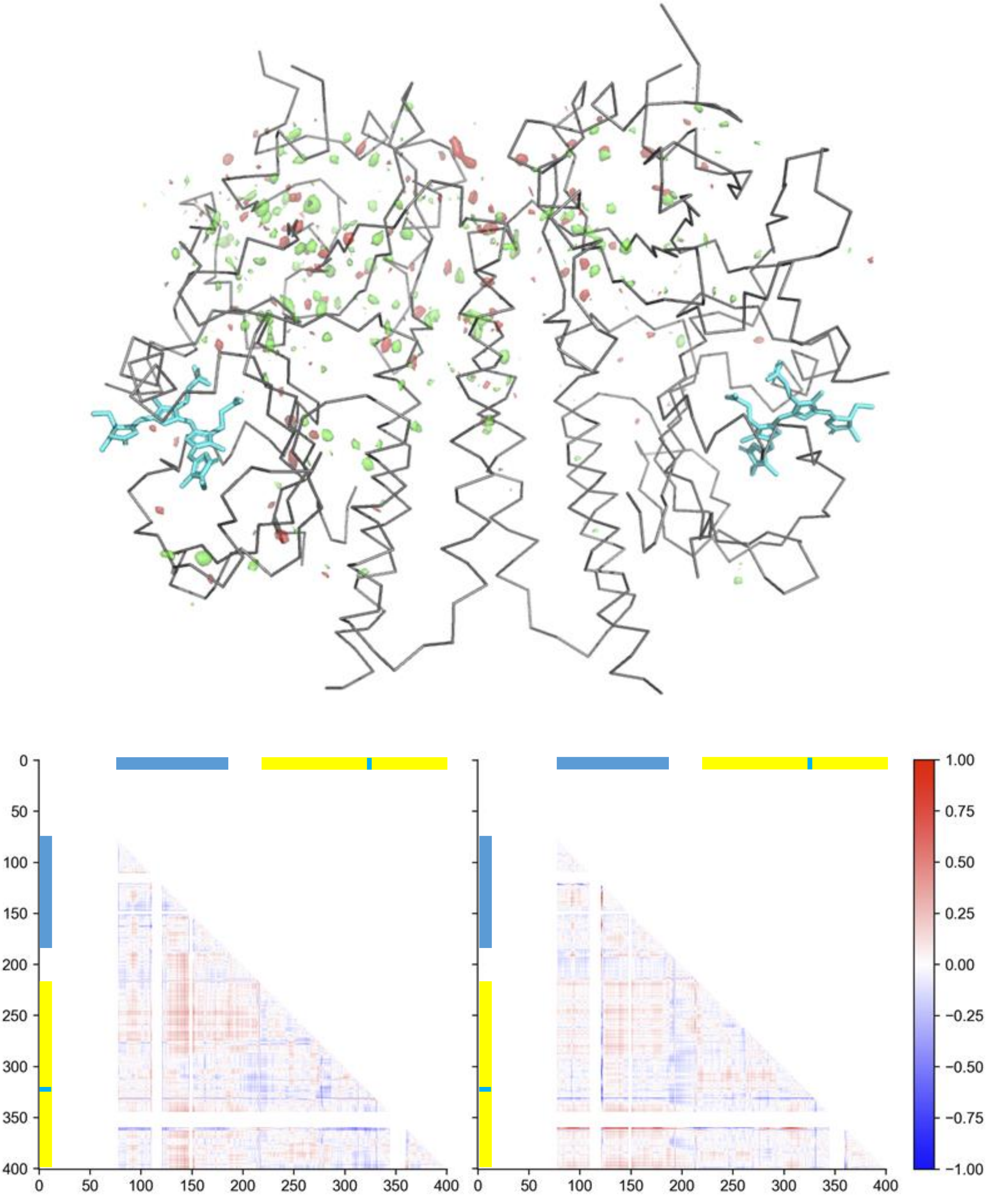

Supplementary Fig. 4 | Comparison of MX structures 8R44 & 8R45 (PCB & PΦB adducts as Pr). Left, chain A; right, chain B. **Above**, Fo-Fo electron density difference map contoured at 3.5 rmsd with main chains and chromophores of 8R44 superimposed. **Below**, Cα difference distance matrix comparisons (colour code, Å relative to 8R44). nPAS & GAF domains, blue & yellow bars; Chromophore, cyan.

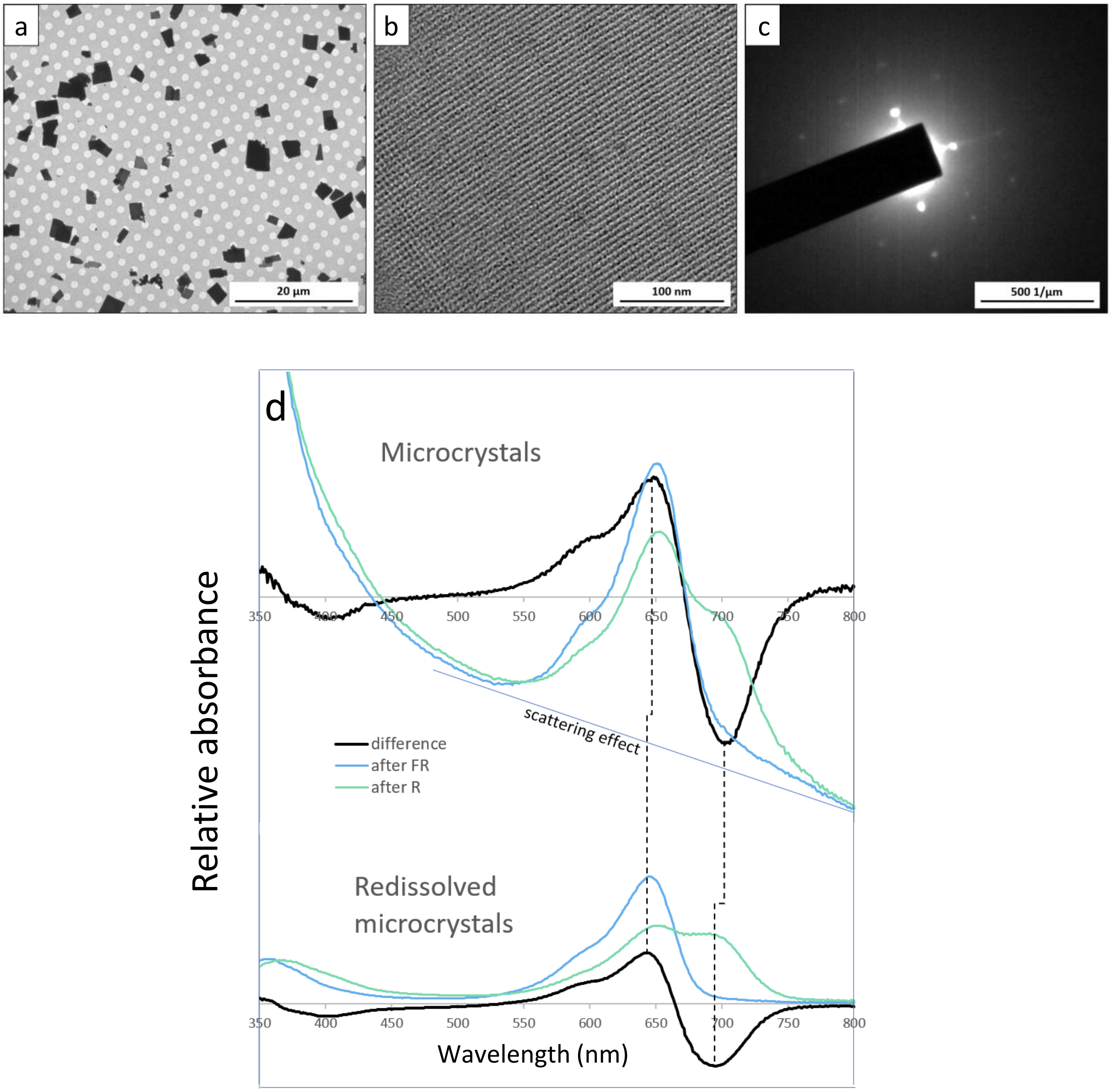

Supplementary Fig. 5 | TEM images and UV-Vis absorption spectra of phyA(nPAS-GAF) microcrystals.

a Thin plate-like crystals with dimensions of 1 - 10 µm on the long edge. **b** Crystal lattice. **c** 1st and 2nd order Bragg diffraction in SAED mode. **d** Absorbance of washed microcrystals and redisolved microcrystals following far-red and red irradiation (cyan and green lines, respectively). The effect of measurement light scattering on the baseline is indicated. The difference spectra (black lines) with maxima (dashed lines) at 648/643 nm and 702/695 nm are shown.

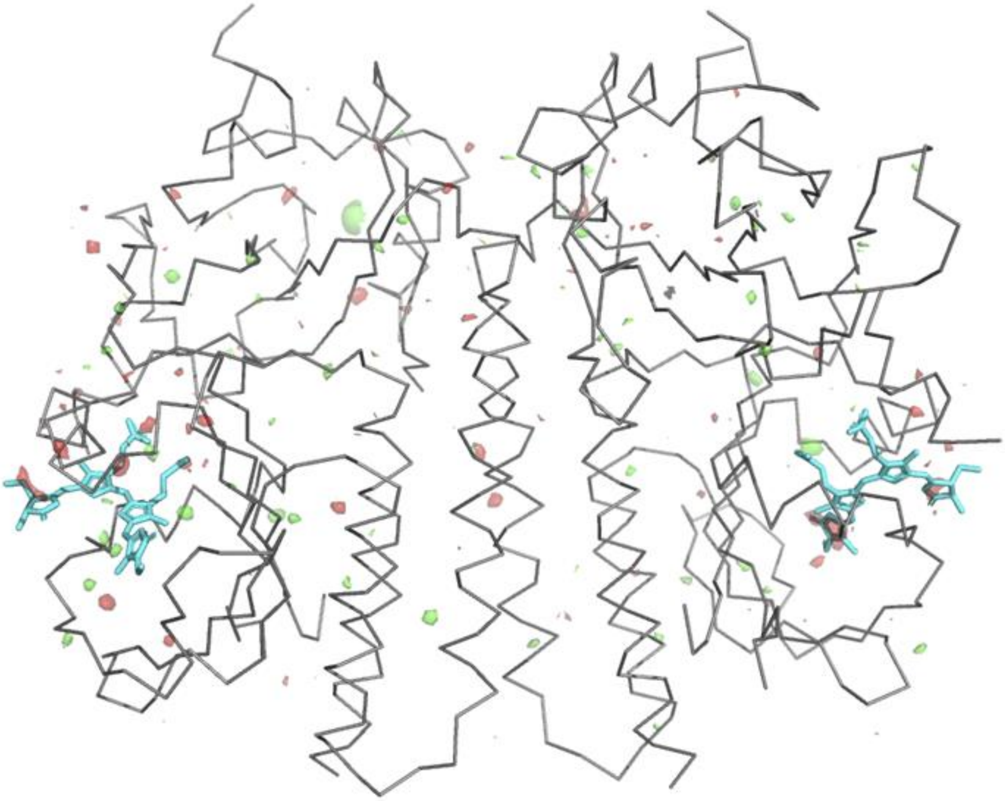

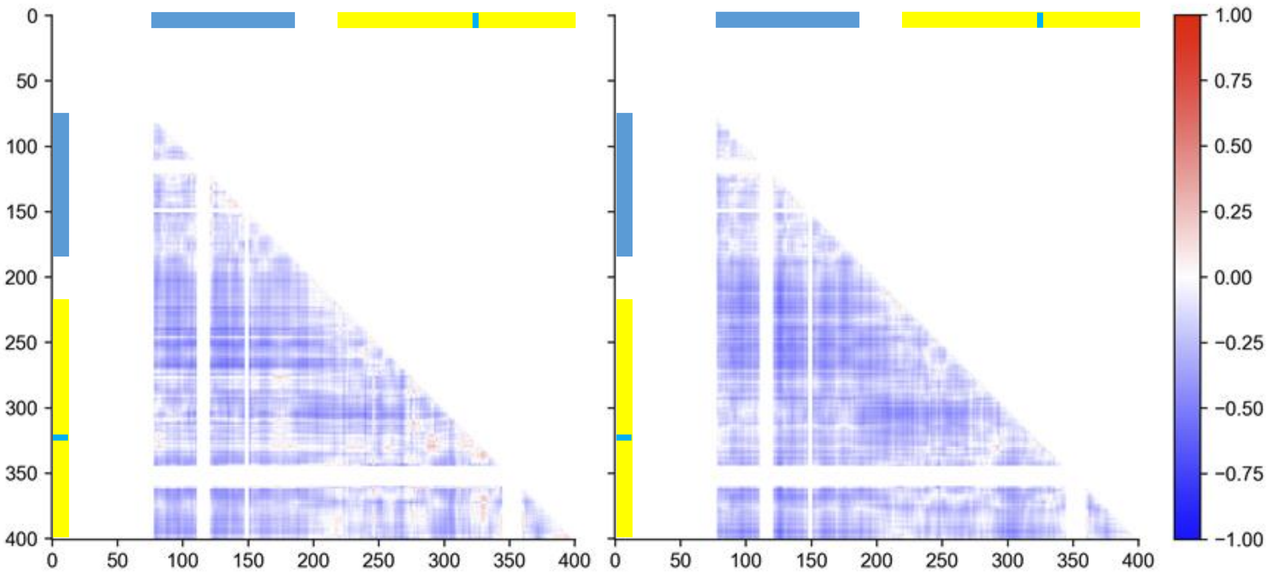

Supplementary Fig. 6 | Comparison of SFX structures 9ER4 and replicate for Pr. Left, chain A; right, chain B. **Above**, Fo-Fo electron difference map contoured at 3.5 rmsd with main chains and chromophores of 9ER4 superimposed. **Below**, Cα difference distance matrix comparisons (colour code, Å relative to 9ER4). nPAS & GAF domains, blue & yellow bars; Chromophore, cyan.

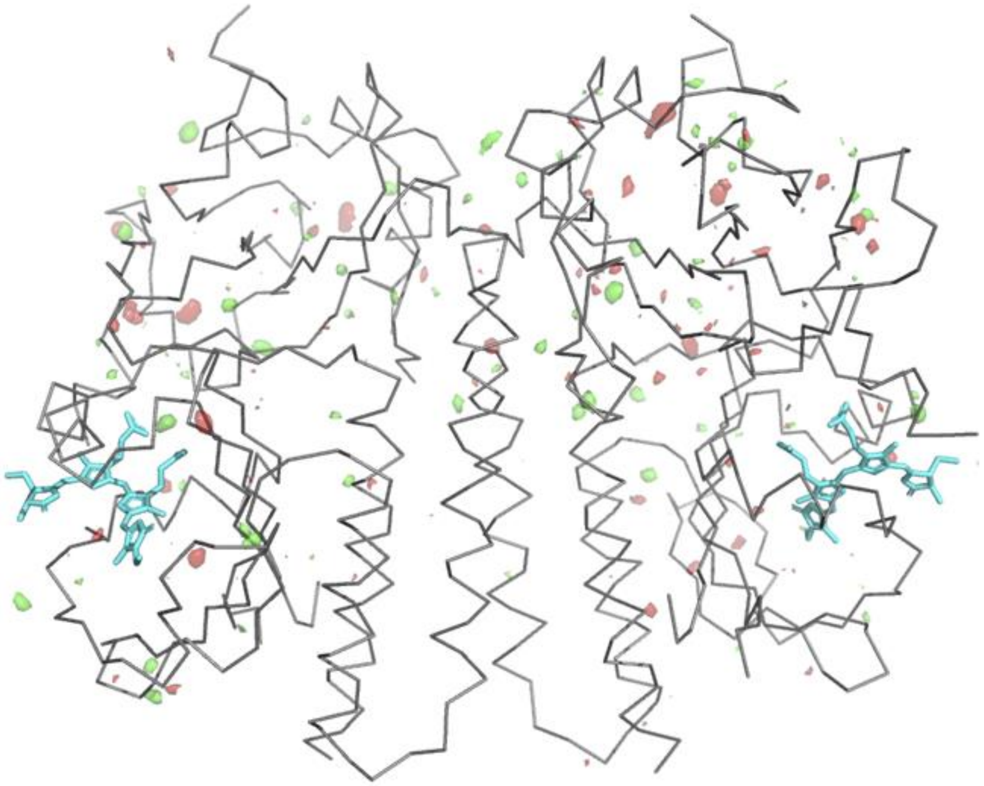

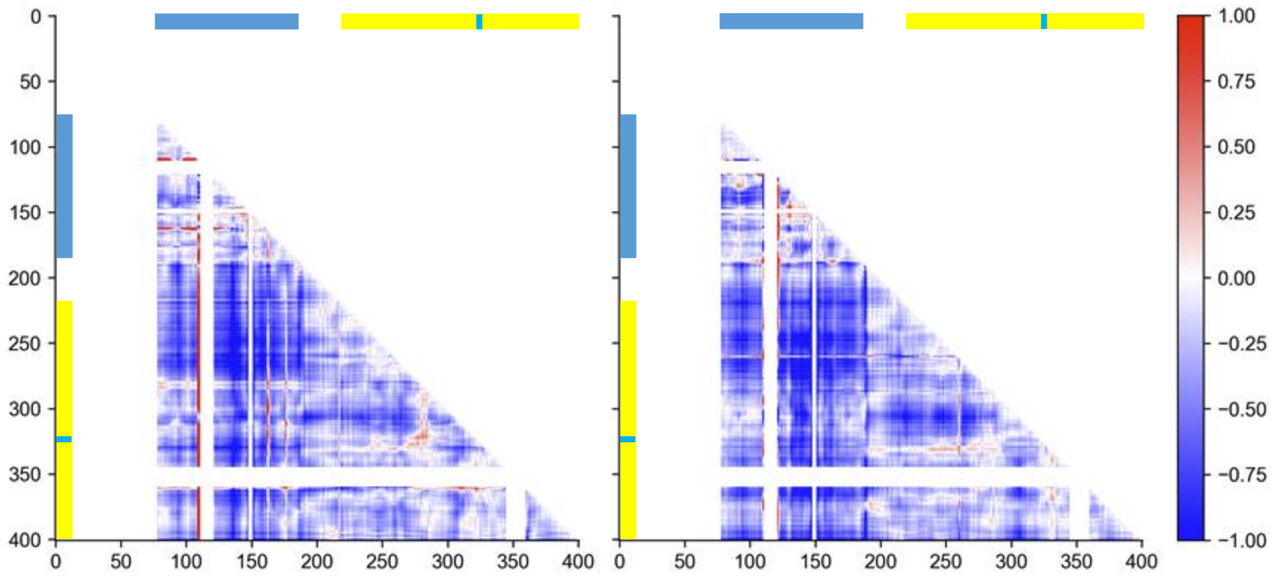

Supplementary Fig. 7 | Comparison of Pr structures 9ER4 *(*SFX) and 8R44 (MX). Left, chain A; right, chain B. **Above**, Fo-Fo electron difference map contoured at 3.5 rmsd with main chains and chromophores of 9ER4 superimposed. **Below**, Cα difference distance matrix comparisons (colour code, Å relative to 9ER4). nPAS & GAF domains, blue & yellow bars; Chromophore, cyan.

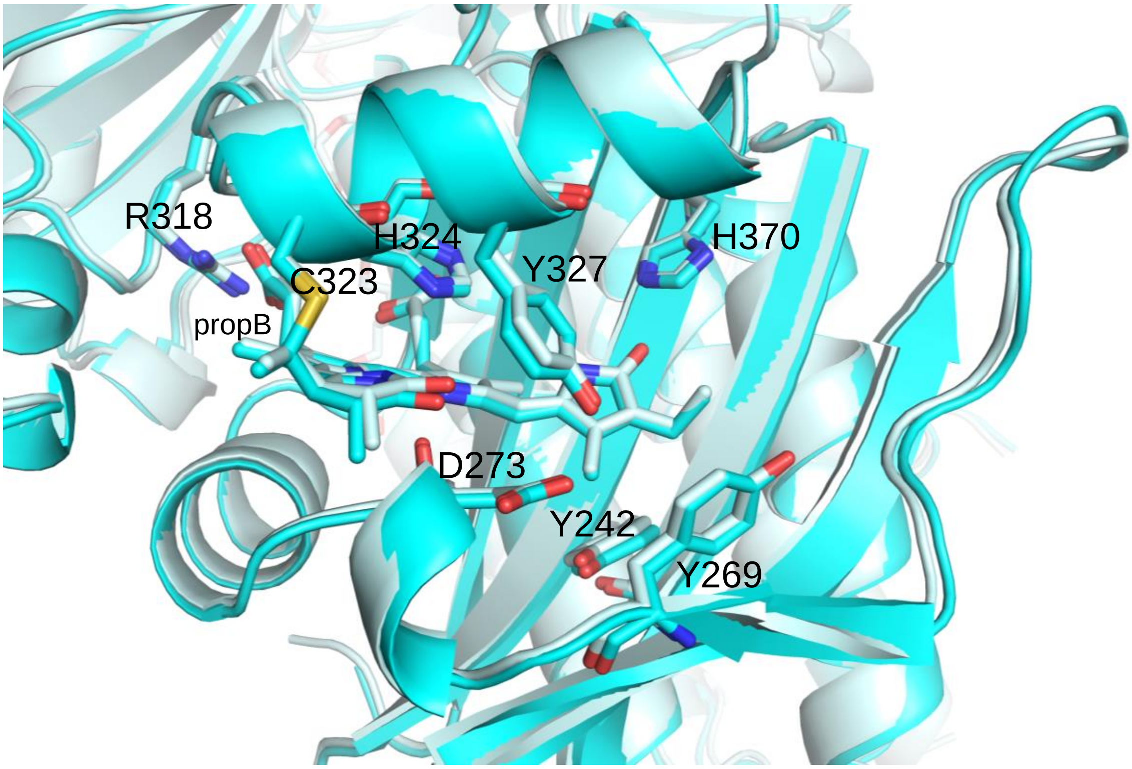

Supplementary Fig. 8 | Comparison of the Pr chromophore pocket in the 8R44 (MX) and 9ER4 (SFX) structures (pale grey and cyan, respectively). Key amino acids and the B-ring propionate are labelled.

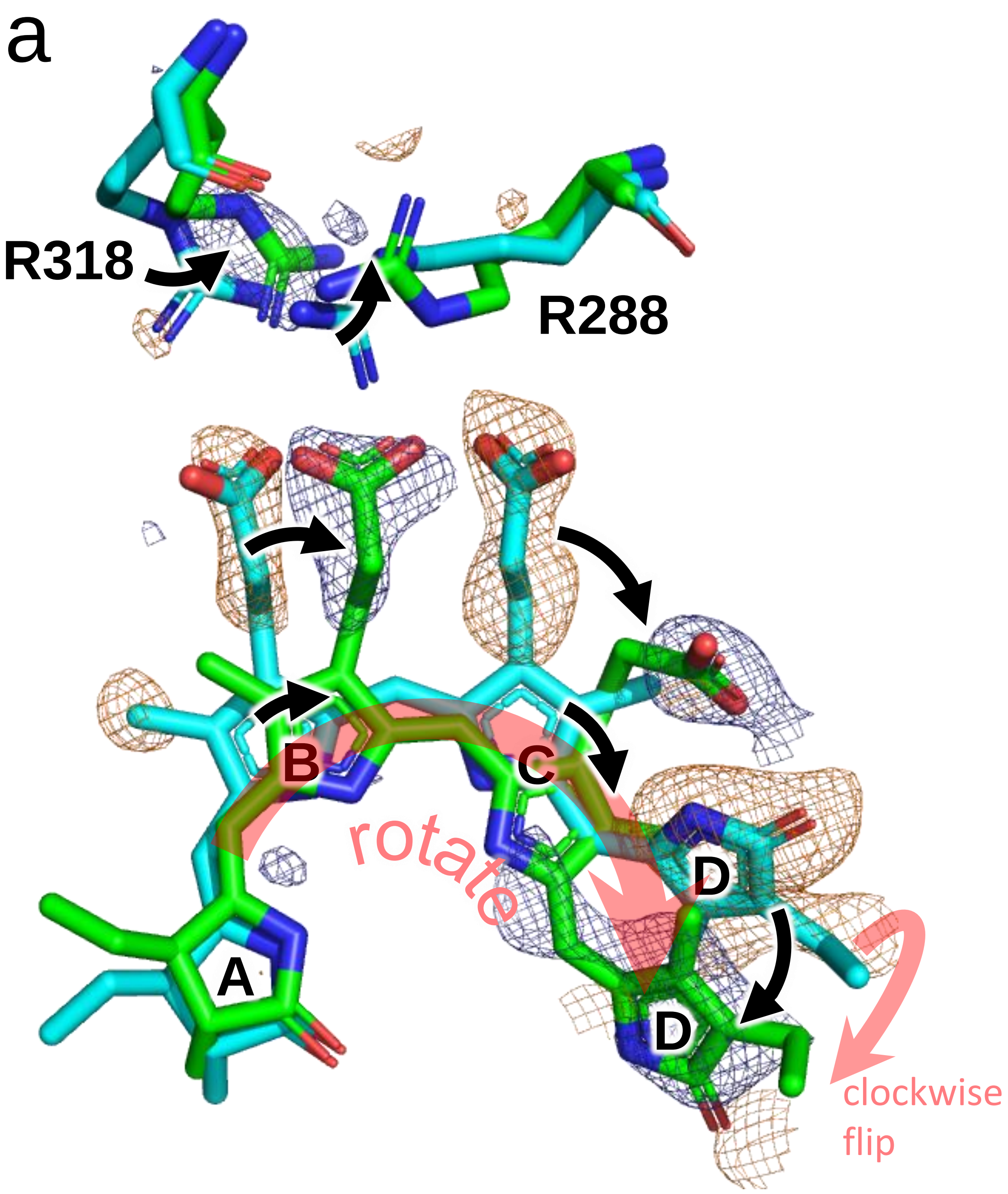

Supplementary Fig. 9a (see next page for legend)

b

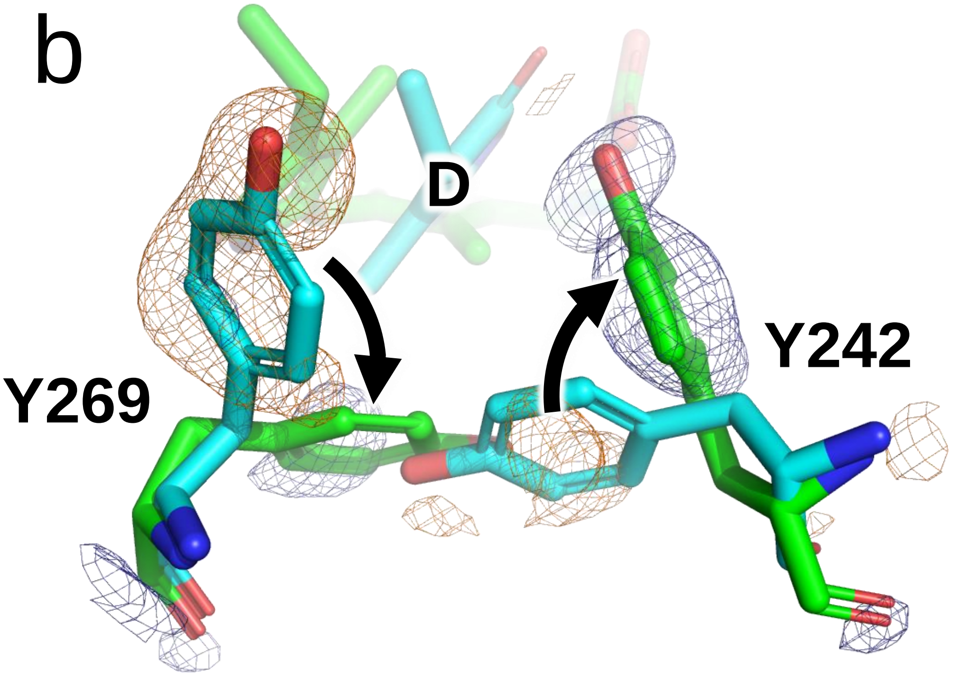

c

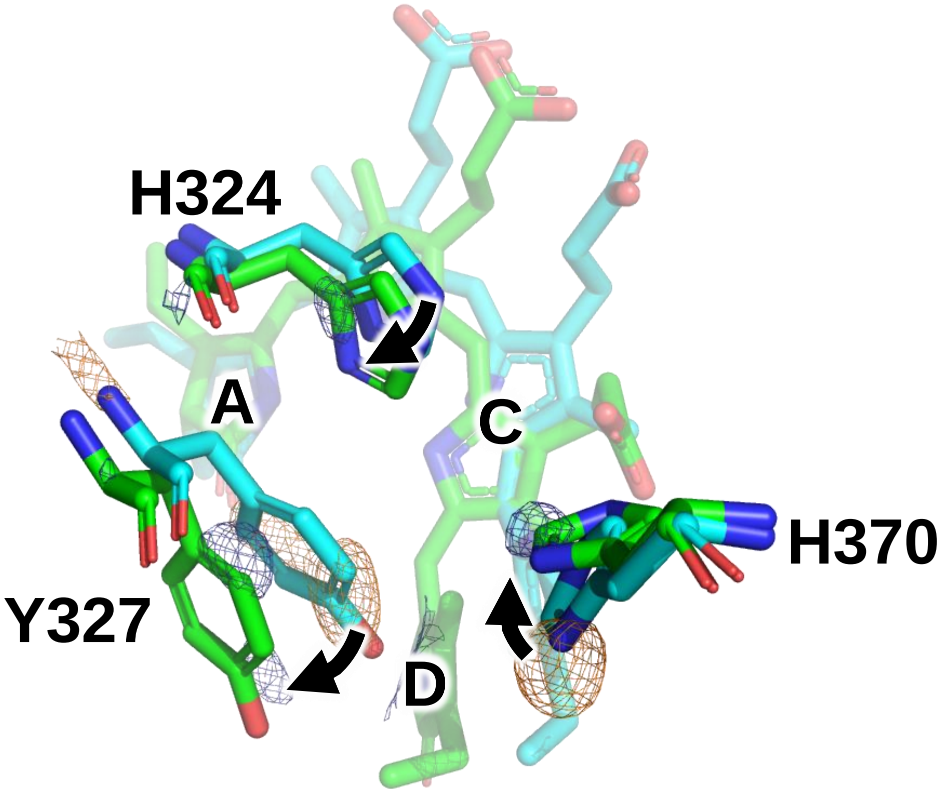

Supplementary Fig. 9 | Evidence for Pr to Pfr conformational changes in the chromophore region from the q-weighted Fo(light)–Fo(dark) difference map. Positive and negative differences are shown as blue and orange mesh, respectively, contoured at +/- 3.0 rmsd. Carbons are shown in cyan (Pr) and green (Pfr). Shifts are highlighted with black arrows. **a** Chromophore and conserved R318 and R288 pair. The flip-and-rotate movements are shown with pink arrows. **b** Chromophore ring D and conserved Y269 and Y242 pair. **c** Chromophore and conserved Y327, H324 and H370. An overall view of the dimer with difference maps is in Fig. 2a.

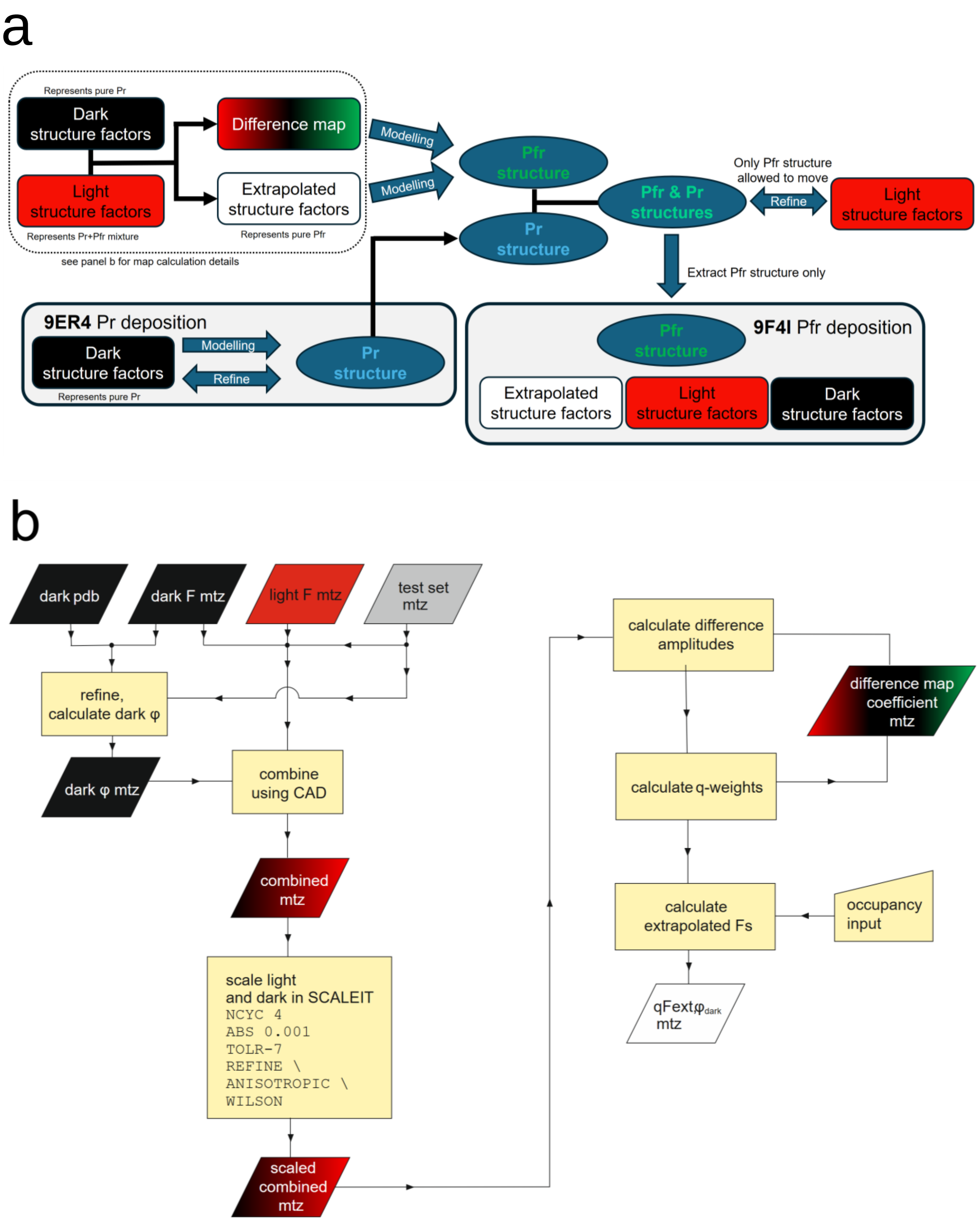

Supplementary Fig. 10 | SFX data processing. a Summary of Pr and Pfr refinement and organisation of deposited SFX data. **b** Calculation of difference- and extrapolated structure factor amplitudes.

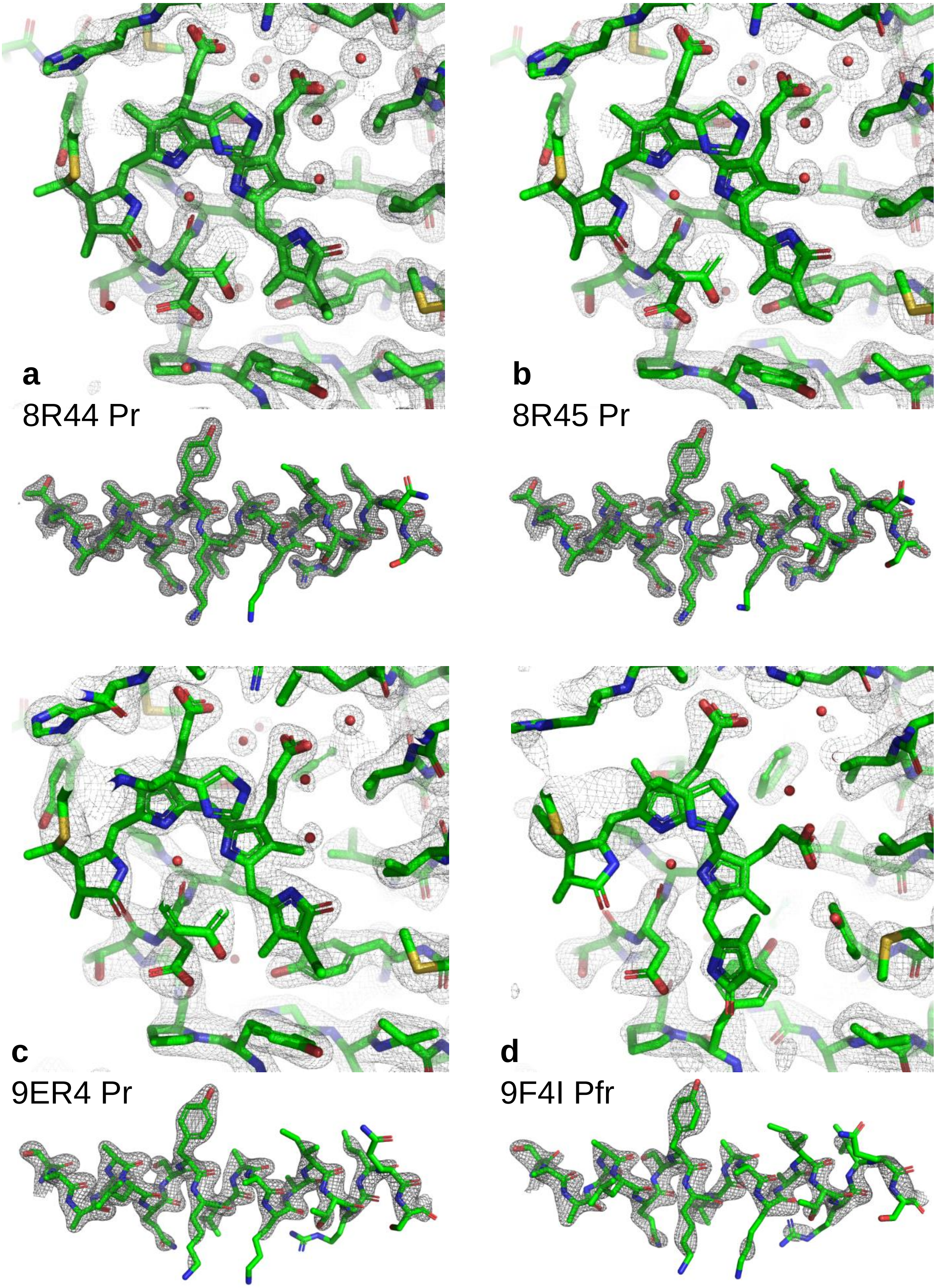

Supplementary Fig. 11 | Electron density maps of the chromophore pocket and representative helical region (residues 193-213). a-c 2Fo-Fc maps of Pr (8R44, 8R45 and 9ER4), **d** extrapolated map of Pfr (9F4I), all for chain B contoured at 1.5 rmsd.

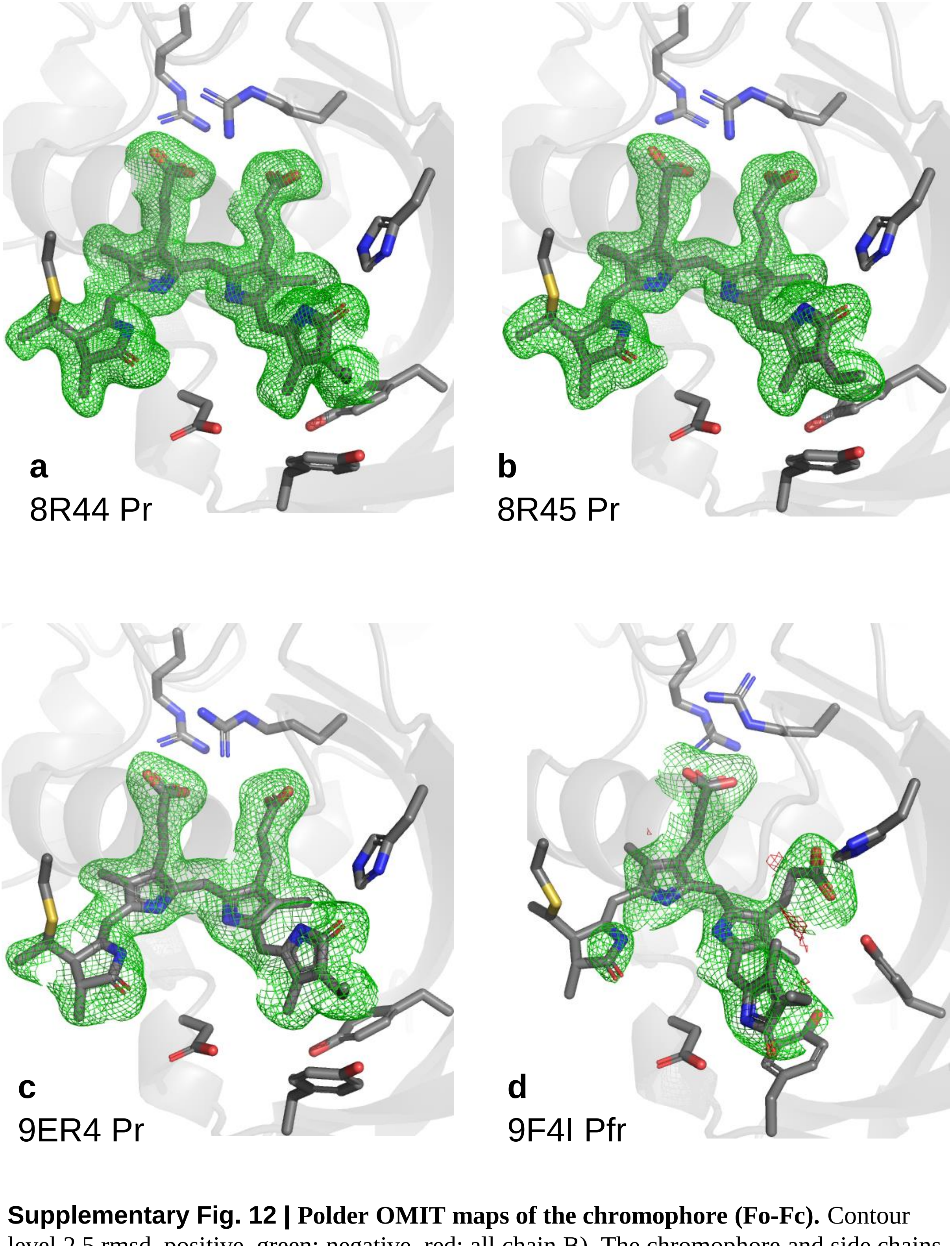

Supplementary Fig. 12 | Polder OMIT maps of the chromophore (Fo-Fc). Contour level 2.5 rmsd, positive, green; negative, red; all chain B). The chromophore and side chains of residues 242, 269, 273, 388, 318, 323 and 370 are shown. The polder OMIT routine in Phenix was chosen due to its higher sensitivity compared to the standard procedure. **a-c** Pr (8R44, 8R45 and 9ER4), **d** Pfr (9F4I).

Supplementary discussion

Dataset and structure comparisons

Our data allowed us to calculate the global Fo-Fo electron density difference maps and Cα difference distance matrices for both protomers of the soybean phyA(nPAS-GAF) crystallographic dimer in order to analyse similarities and differences. To provide a rigorous background for our description of the structural effects of photoactivation, we also compared structural differences independent of photochemistry. Here we compare PCB and PΦB adduct structures from the BESSY II synchrotron (single crystals under cryogenic conditions), PCB adduct structures from independent experiments at European XFEL (SFX with microcrystals at ambient temperature), and PCB adduct structures from the BESSY II synchrotron and European XFEL. We also show details of the microcrystals.

At ambient temperature, Pfr→Pr thermal reversion of PΦB adducts of the construct is approximately twice as rapid as for PCB adducts (Supplementary Fig. 2). Analysis of the PCB (8R44) and PΦB (8R45) adduct structures in the Pr state (Supplementary Figs. 3 and 4) reveals a slight global shift (rmsd = 0.25 Å for all Cα atoms in the dimer), mostly resulting from a *ca*. 0.5 Å movement of nPAS in protomer A. Water 518 in the 8R44 structure is not apparent from the electron density map of 8R45 while waters 396 and 306 are only weakly represented, perhaps reflecting greater mobility in the case of the PΦB adduct. Otherwise, the only obvious difference is the expected coplanarity of the vinyl group with ring D. This implies that the thermal reversion rate is influenced by an effect of the different D-ring side chains on mobility. Whereas crystal structures present an essentially static view, MAS NMR has shown clearly that Pr comprises at least two sub-states (Pr-I and Pr-II)[1,2] whereas Raman spectroscopy implies two, subtly different chromophore configurations in Pfr[3]. Presumably, the rate of thermal reversion is governed by the activation energy barrier. However, the more rigid vinyl would be more likely to have stronger steric effects on nearby moieties and thereby raise rather than lower the barrier. Alternatively, the vinyl might interact to destabilise Pfr itself, although it is unclear from the 9F4I structure what interactions might be involved.

Supplementary Fig. 5 shows TEM images following fixation and negative staining with uranyl acetate and UV-Vis absorption spectra of thin, plate-like microcrystals of phyA(nPAS-GAF). The crystal lattice is readily visible, as is first- and second-order Bragg diffraction (despite the fixation process). The native microcrystals show clear red/far-red

photochromicity (slightly red-shifted relative to the solution state, as also apparent from the difference maxima at 648/643 nm and 702/695 nm), demonstrating functionality.

Supplementary Fig. 6 illustrates the minimal differences between replicate SFX datasets for Pr at ambient temperature. Superimposition of the final refined SFX structures yielded an initial rmsd of 0.58 Å for all protein atoms and 0.23 Å for all Cα atoms in the dimer, consistent with the global Fo-Fo and difference distance matrix analyses, respectively.

Supplementary Fig. 7 illustrates the differences between the SFX ambient temperature and MX cryogenic datasets for Pr (8R44 and 9ER4, respectively). Superimposition yielded an rmsd of 0.4 Å for all Cα atoms in the dimer, consistent with the difference distance matrices. The distances of each Cα atom from the centre of mass of the crystallographic dimer were measured using PyMol and the temperature-related differences calculated, yielding a mean linear expansion coefficient of 49 x 10^{-6} / K, typical of proteins *in crystallo*. At the level of the refined models, local superimpositions show the structures to be almost identical (e.g. Supplementary Fig. 8). The thermal effect on the dimer dimensions is *ca*. 1% and therefore not evident in the Fo-Fo map (Supplementary Fig. 7), but this nevertheless reveals numerous small difference areas that do not correspond to a consistent structural change such as water or side chain movements.

Supplementary Fig. 9 shows the q-weighted Fo(light)-Fo(dark) difference maps overlayed on the final, refined Pr / Pfr structures in the chromophore region of protomer B.

Supplementary methods

Calculation of difference- and extrapolated maps

Supplementary Fig. 10. Panel **a** shows the structure solution process for the SFX datasets. Data from dark-adapted and red-light-irradiated crystals are used to calculate difference map coefficients as well as extrapolated structure factor amplitudes. These are used to model and refine the mixture of Pr- and Pfr-state structures that is deposited as PDB entry 9QZT, together with the various data sets. The dark-adapted data alone are used to model and refine the dark-state Pr structure, deposited as entry 9ER4. Panel **b** shows the details of the calculation of difference- and extrapolated structure factor amplitudes to derive the photoactivated Pfr structure deposited as 9F4I, as explained below.

Irradiated-dark difference maps and extrapolated structure factor maps [4,5] were calculated using a script (available at https://github.com/tbarends/SFextrapolation) that works as follows (Supplementary Figure 10b). The user provides the names of the mtz files containing the structure factor amplitudes of the dark-adapted and red-light-irradiated crystals ($|\overrightarrow{F_{dark}}|$ and $|\overrightarrow{F_{light}}|$) and their sigmas ($\sigma(|\overrightarrow{F_{dark}}|)$ and $\sigma(\overrightarrow{F_{light}})$), as well as an mtz file containing test-set labels. The user also enters the name of a pdb file containing the model to be used for phasing, and sets the occupancy to be used. The user also selects whether q-weighting[6] is to be employed (as in the case for the current project), and a resolution range (20-2.2 Å for the current project).

The script begins by determining the phases for the dark-adapted sample (Pr). To this end, 10 cycles each of rigid-body and restrained refinement of the model against the dark-adapted structure factor amplitudes are performed using REFMAC5[7]. Then, these phases are combined with the dark-adapted amplitudes, the amplitudes from the red-light-irradiated sample and the test-set flags into a single mtz file using CAD[8]. This file is then supplied to SCALEIT[9] to scale irradiated- and dark-adapted amplitudes together. During scaling, an anisotropic temperature factor is applied, as well as a final Wilson scaling step (“REFINE ANISOTROPIC WILSON”). Convergence criteria were set to a minimum of four cycles, all parameter shifts should be less than 0.001 standard deviations and a tolerance of 10^{-7} was employed (“CONVERGENCE NCYC 4” “CONVERGENCE ABS 0.001” and “CONVERGENCE TOLR -7”).

The mtz file with the scaled amplitudes is then read into a Python program that uses SFTOOLS to add the multiplicity and a flag indicating whether a reflection is centric or not to each h,k,l combination. This script then calculates difference amplitudes and their standard deviations:

$$\Delta F = |\overrightarrow{F_{light}}| - |\overrightarrow{F_{dark}}|$$

$$\sigma(\Delta F) = \sqrt{\left(\sigma(|\overrightarrow{F_{light}}|)\right)^2 + \left(\sigma(|\overrightarrow{F_{dark}}|)\right)^2}$$

Then, Q-weighting is applied to these differences, as described in[6]. Briefly, the data are divided into resolution bins (20 in the case described here), and the average of the parameter

σ_D for each bin is estimated as $\langle\sigma_D^2\rangle = \langle\frac{n\left(\left|\overrightarrow{F_{light}}\right|-\left|\overrightarrow{F_{dark}}\right|\right)^2}{\varepsilon}\rangle$ where ε is the multiplicity of each reflection and n is 1 for acentric and 2 for centric reflections. $\langle\sigma_D^2\rangle$ is an estimate of the average light-dark differences, but for noisy data is contaminated with measurement errors that typically increase with resolution. The influence of these errors is removed as much as possible by deconvolution; the average $\langle\left(\sigma\left(\left|\overrightarrow{F_{light}}\right|\right)\right)^2 - \left(\sigma\left(\left|\overrightarrow{F_{dark}}\right|\right)\right)^2\rangle$ is subtracted from the $\left(\left|\overrightarrow{F_{light}}\right| - \left|\overrightarrow{F_{dark}}\right|\right)$ term in the calculation of $\langle\sigma_D^2\rangle$ while ensuring that $\langle\sigma_D^2\rangle$ never becomes less than $\frac{1}{2}\langle\frac{n\left(\left|\overrightarrow{F_{light}}\right|-\left|\overrightarrow{F_{dark}}\right|\right)^2}{\varepsilon}\rangle$ for any particular bin.

The resulting estimates of σ_D^2 are then used to calculate the weighting factors q for each reflection:

$$q_a = \frac{(\varepsilon\sigma_D^2/2)}{\left(\sigma\left(\left|\overrightarrow{F_{light}}\right|\right)\right)^2 + \left(\sigma\left(\left|\overrightarrow{F_{dark}}\right|\right)\right)^2 + (\varepsilon\sigma_D^2/2)}$$

for acentric reflections and

$$q_c = \frac{\varepsilon\sigma_D^2}{\left(\sigma\left(\left|\overrightarrow{F_{light}}\right|\right)\right)^2 + \left(\sigma\left(\left|\overrightarrow{F_{dark}}\right|\right)\right)^2 + \varepsilon\sigma_D^2}$$

(Equation 31 in [6], without the use of the figure of merit as discussed therein).

The difference amplitudes ΔF are then weighted with the q-factors:

$$\Delta F_{hkl} = \frac{q_{hkl}}{\langle q\rangle}\Delta F_{hkl}$$

The script then writes out an mtz file with the q-weighted difference amplitudes and model phases for map calculation and display.

Finally, extrapolated structure factors are calculated using the q-weighted differences, the dark-state amplitudes, and the preset occupancy estimate o:

$$F_{extrapolated,hkl} = \frac{\frac{q_{hkl}}{\langle q\rangle}\Delta F_{hkl}}{o} + \left|\overrightarrow{F_{dark}}\right|$$

estimating the error as $\sigma\left(F_{extrapolated,hkl}\right) = \sqrt{(\sigma(\Delta F)/o)^2 + \left(\sigma\left(\left|\overrightarrow{F_{dark}}\right|\right)\right)^2}$

These amplitudes are exported as an MTZ file together with the dark-state model phases and test set flags for map display and refinement.

The script also contains other functions, such as automated occupancy estimation and the calculation of various maps, but these were not used for the current project.

Construction of the structural models

The most striking features in the q-weighted difference electron density maps in both subunits are negative peaks on the cofactor carboxylate groups combined with positive peaks adjacent to these groups (Supplementary Fig. 9a), indicating a shift of the carboxylates towards the positive peaks. The q-weighted extrapolated densities are consistent with this, and the most parsimonious explanation of the observed densities is a rotation of the cofactor around an axis perpendicular to its plane as indicated in Fig. 4 and Supplementary Fig. 9a). Also striking in both subunits are a negative peak at the D-ring in combination with a positive feature in the shape of the D-ring and its connection to the C-ring close to it, consistent with the expected D-ring flip. The structure was modeled as such (Fig. 4 and Supplementary Fig. 9a) using the extrapolated density for the positioning of the D-ring and ethylidine bridge to ring C. In the same way and again in both subunits, negative peaks for Y269 and Y242 in combination with positive peaks close to them indicate a change in conformation for these residues which was modeled accordingly (Fig. 5d and Supplementary Fig. 9b). Smaller features around R318/R288 (Fig. 5a and Supplementary Fig. 9c) as well as H324, H370 and Y327 (Fig. 5c and Supplementary Fig. 9c) showed changes in the conformation of these residues in both subunits as well, which were also modeled along with numerous smaller structural changes.

The preliminary Pfr state model thus prepared was then subjected to ensemble refinement. To that end, the occupancies of the two polypeptide chains of the Pfr model were set to the estimated occupancies (0.11 and 0.23 for chain for chains A and B, respectively). The resulting PDB file was then combined with that of the Pr state, in which the occupancies of the A and B chains were set to 0.89 and 0.77, respectively. The resulting structure was then refined against the original (*i.e.*, not-extrapolated) structure factors using PHENIX[10,11] while allowing only the Pfr part of the structure to move.[12] Occupancy refinement was not performed.

Supplementary Figs. 11 and 12 show selected 2Fo-Fc electron density and Fo-Fc polder omit maps, respectively, of all four final structures. The polder omit algorithm in Phenix was used to avoid the noise that is introduced by standard procedures which would insert waters into the space left when the chromophore is omitted[10,13].

References

1 Song, C. *et al.* 3D structures of plant phytochrome A as Pr and Pfr from solid-state NMR: Implications for molecular function. *Front Plant Sci* **9**, 498, doi:10.3389/fpls.2018.00498 (2018).

2 Song, C. *et al.* Two ground state isoforms and a chromophore *D*-ring photoflip triggering extensive intramolecular changes in a canonical phytochrome. *Proc Natl Acad Sci USA* **108**, 3842-3847, doi:10.1073/pnas.1013377108 (2011).

3 Velazquez Escobar, F. *et al.* Structural communication between the chromophore-binding pocket and the N-terminal extension in plant phytochrome phyB. *FEBS Lett.* **591**, 1258-1265, doi:10.1002/1873-3468.12642 (2017).

4 Genick, U. K. Structure-factor extrapolation using the scalar approximation: theory, applications and limitations. *Acta Crystallogr D Biol Crystallogr* **63**, 1029-1041, doi:10.1107/S0907444907038164 (2007).

5 Genick, U. K. *et al.* Structure of a protein photocycle intermediate by millisecond time-resolved crystallography. *Science* **275**, 1471-1475 (1997).

6 Ursby, T. & Bourgeois, D. Improved estimation of structure-factor difference amplitudes from poorly accurate data. *Acta Crystallogr A* **53**, 564-575, doi:Doi 10.1107/S0108767397004522 (1997).

7 Murshudov, G. N. *et al.* REFMAC5 for the refinement of macromolecular crystal structures. *Acta Crystallographica Section D* **67**, 355-367, doi:10.1107/S0907444911001314 (2011).

8 Agirre, J. *et al.* The CCP4 suite: integrative software for macromolecular crystallography. *Acta Crystallographica Section D: Structural Biology* **79**, 449-461 (2023).

9 Howell, L. & Smith, D. Normal Probability Analysis. *Journal of applied crystallography* **25**, 81-86 (1992).

10 Liebschner, D. *et al.* Macromolecular structure determination using X-rays, neutrons and electrons: recent developments in Phenix. *Acta crystallographica. Section D, Structural biology* **75**, 861-877, doi:10.1107/S2059798319011471 (2019).

11 Adams, P. D. *et al.* PHENIX: a comprehensive Python-based system for macromolecular structure solution. *Acta Crystallographica Section D: Biological Crystallography* **66**, 213-221 (2010).

12 Barends, T. R. M. *et al.* Influence of pump laser fluence on ultrafast myoglobin structural dynamics. *Nature* **626**, 905-911, doi:10.1038/s41586-024-07032-9 (2024).

13 Liebschner, D. *et al.* Polder maps: improving OMIT maps by excluding bulk solvent. *Acta crystallographica. Section D, Structural biology* **73**, 148-157, doi:10.1107/S2059798316018210 (2017).
